# Association of *ACTN3* Polymorphism with Body Somatotype and Cardiorespiratory Fitness in Young Healthy Adults

**DOI:** 10.3390/ijerph16091489

**Published:** 2019-04-27

**Authors:** Natalia Potocka, Beata Penar-Zadarko, Marzena Skrzypa, Marcin Braun, Maria Zadarko-Domaradzka, Mariusz Ozimek, Edyta Nizioł-Babiarz, Zbigniew Barabasz, Izabela Zawlik, Emilian Zadarko

**Affiliations:** 1 Laboratory of Molecular Biology, Centre for Innovative Research in Medical and Natural Sciences, Faculty of Medicine, University of Rzeszow, 35-959 Rzeszow, Poland; npotocka@ur.edu.pl (N.P.); mskrzypa@ur.edu.pl (M.S.); 2 Institute of Nursing and Health Sciences, Faculty of Medicine, University of Rzeszow, 35-959 Rzeszow, Poland; bpenar@ur.edu.pl; 3 Innovative Research Laboratory in Nursing, Centre for Innovative Research in Medical and Natural Sciences, Faculty of Medicine, University of Rzeszow, 35-959 Rzeszow, Poland; 4 Department of Pathology, Chair of Oncology, Medical University of Lodz, 90-419 Lodz, Poland; braunmarcin@gmail.com; 5 Postgraduate School of Molecular Medicine, Medical University of Warsaw, 02-091 Warsaw, Poland; 6 Department of Human Sciences, Faculty of Physical Education, University of Rzeszow, 35-959 Rzeszow, Poland; mzadarko@ur.edu.pl; 7 Institute of Sport-National Research Institute, 02-091 Warsaw, Poland; mozime@poczta.onet.pl; 8 Department of Health Sciences, Faculty of Physical Education, University of Rzeszow, 35-959 Rzeszow, Poland; edyta.niziol@gmail.com (E.N.-B.); zbarabasz@ur.edu.pl (Z.B.); ezadarko@ur.edu.pl (E.Z.); 9 Department of Genetics, Institution of Experimental and Clinical Medicine, Faculty of Medicine, University of Rzeszow, 35-959 Rzeszow, Poland

**Keywords:** *ACTN3*, polymorphism, health-related fitness, HRmax, HRM PCR, sport genetic

## Abstract

*ACTN3* encodes the protein α-actinin-3, which affects the muscle phenotype. In the present study, we examined the association of *ACTN3* R577X polymorphism with body somatotype and cardiorespiratory fitness in young, healthy adults. The study group included 304 young adults, in whom cardiorespiratory fitness was evaluated and the maximum oxygen uptake was determined directly. The somatotype components were calculated according to the Heath-Carter method. Genotyping for the *ACTN3* gene was performed using a polymerase chain reaction followed by high-resolution melting analysis. In the female group, a lower maximal heart rate (HRmax) was more strongly associated with the RR genotype (*p* = 0.0216) than with the RX and XX genotypes. In the male group, the *ACTN3* RX genotype, as compared with other genotypes, tended to be associated with a lower percentage of adipose tissue (*p* = 0.0683), as also reflected by the body mass index (*p* = 0.0816). *ACTN3* gene polymorphism may affect cardiorespiratory fitness. Our analysis of *ACTN3* gene polymorphism does not clearly illustrate the relationships among genotype, body composition, and somatotype in young, healthy adults.

## 1. Introduction

Over the past several decades, research has focused on the influence of genes on physical fitness. Genes affecting health-related fitness can be divided into groups that are responsible for muscle energy efficiency, muscle endurance, cardiorespiratory fitness, susceptibility to injury, and psychological conditions. This categorization led to the identification of numerous genes that may aid in differentiating between elite and non-elite athletes.

An important gene involved in physical fitness genetics is *ACTN3*, a 16406-bp gene located on chromosome 11q13-q14. *ACTN3* is referred to as “a gene for speed” [1,2]. *ACTN3* encodes α-actinin-3, a member of the highly conserved family of α-actinin proteins. The α-actinin-3 protein contains 901 amino acids and is an actin-binding protein with many roles in different cell types. *ACTN3* gene expression is limited to skeletal muscle. It is located in the Z-disc and analogous dense bodies, where it aids in anchoring the myofibrillar actin filaments [3]. In intensely contracting fibres, Z-discs are exposed to trauma caused by training; therefore, the basic function of α-actinin-3 is thought to be the minimization of damage caused by eccentric muscle contractions [4]. The α-actinin-3 protein is present only in type II fibres, which contract most rapidly during short, high-intensity activities such as sprinting [5].

Genetic analysis of the *ACTN3* gene revealed a polymorphism, R577X (rs1815739), that yields a premature stop codon resulting in a non-functional α-actinin-3 protein due to the transversion of cytosine to thymine [2]. The allele with an arginine codon is denoted R, and the allele with a non-sense mutation is denoted X. In the human population, it is possible to have RR genotypes (homozygous with functional protein), RX (heterozygous, with normal protein), and XX (homozygous, with α-actinin-3 deficiency). This polymorphism, described by North et al. in 1999 [6], occurs in 18% of the human population [7]. In 2003, Yang et al. reported that the R577X polymorphism of the *ACTN3* gene correlated with endurance sports. Subsequent research indicates that the *ACTN3* gene determines parameters such as human speed, endurance, and performance [4,8,9].

The ability of athletes in speed or endurance contests is determined by the ability of muscle to adapt with training. There are two types of muscle fibres, which are detectable on the basis of colour. Human muscles are heterogeneous: the fibres that compose them include different types in various proportions, forming a cross-sectional mosaic muscle. Red muscle fibres (fibre type I or slow-twitch fibres), due to the high storage capacity of the blood and the high percentage of myoglobin and mitochondria, appear red under light microscopy. The fibres slowly become exhausted and use glycogen and fat as their fuel. White muscle fibres (type II fibres or fibres with fast contraction) have an average blood storage capacity, and low levels of myoglobin and mitochondria. They use only glycogen as their fuel, and, compared with red fibres, they become exhausted more quickly; however, they are larger than red fibres and have stronger contractions [2,6]. Homozygotes with two X alleles are incapable of producing α-actinin-3, thus potentially altering the function of type II muscle fibre; however, no disorders in muscle formation have been observed in people with this genotype [6,10]. The α-actinin-3 protein is present only in type II fibres, that is, those that contract most rapidly and are utilized most intensively during short, high-intensity activities such as sprinting in individuals with RX or RR genotypes. A relationship between the R allele and the RR genotype and the outstanding power performance of athletes with different ancestries has been demonstrated [11,12,13]. Vincent et al. reported that the proportion of fast-twitch muscle fibres is greater in healthy young males with the RR genotype than in those with the XX genotype [14]. The R allele occurs at high frequency in outstanding athletes [4]. Other studies noted that the occurrence of two R alleles is related to muscle strength and the distribution of muscle fibres [14,15].

The aim of this study was to investigate the association between *ACTN3* gene polymorphism and body composition, somatotypes, and cardiorespiratory fitness in young, healthy adults.

## 2. Materials and Methods

### 2.1. Study Population

The study was cross-sectional, and the project was approved by the Bioethics Committee of the University of Rzeszow no. 20/12/2015. The study involved 1097 healthy students (487 males and 610 females) studying in major academic centres in Poland (University of Rzeszow, Cracow University of Technology, University of Life Sciences in Poznan, Maria Curie-Sklodowska University in Lublin). The group selection occurred in two stages. The first stage included randomly selected 1st degree students who had been tested for cardiorespiratory fitness with a 20 m shuttle run test (20 m SRT). The 20 m SRT was conducted as described by Leger et al. in 1988 [16]. To achieve the highest possible variation of results, in the second stage, participants who completed the 20 m SRT below and above the median distance (laps) were randomly selected on the basis of the median distance (laps) of the surveyed males (median = 1520 m, 76 laps) and females (median = 880 m, 44 laps). In the second stage, 309 people (155 males and 154 females) participated in a 20 m SRT. The aim of this stage was to define the maximum oxygen uptake (VO_2_max) with a portable gas analyser K4b^2^ (Cosmed, Roma, Italy). The K4b^2^ was calibrated in accordance with the manufacturer’s specifications at the beginning of each test day. The speed was 8.5 km/h during the first minute and increased by 0.5 km/h every minute. Throughout the test, the heart rate (HR) was recorded every 5 s with a Fix Polar Heart Rate Transmitter Belt (Polar Electro Oy, Kempele, Finland). Before the cardiac stress test, the trained research team performed anthropometric measurements. Body height was measured with a stadiometer (SECA 213, Hamburg, Germany) with an accuracy of 1 mm. Body weight and composition, including fat mass (fat% and fat kg), free fat mass, and total body water, were measured using the electrical bioimpedance method with a body composition analyser (Tanita TBF 300, Tokyo, Japan). Measurements of the waist and hip circumferences were determined with an inflexible elastic centimetre tape. The waist circumference was measured according to the protocol of the World Health Organization [17]. The World Health Organization STEPwise Approach to Surveillance protocol comprises approximating the midpoint between the lower margin of the last palpable rib and the top of the iliac crest. The hip circumference was measured around the widest portion of the buttocks. The body mass index (BMI), waist hip ratio (WHR), and waist height ratio (WHtR) were determined. The somatotype components of the individual subjects were calculated according to the Heath-Carter method [18]. The somatotype describes the morphological characteristics of the body, demonstrating the specificity of the structure and shape of the body; it depends on the sex, age, height, body composition, and lifestyle (nutrition, physical activity, and smoking status) [19]. The current morphological state of the subject may be expressed by the level of development of the following three components: endomorphy (relative fatness), mesomorphy (relative musculoskeletal robustness), and ectomorphy (relative linearity or slenderness of a physique) [20,21]. The genetics testing was performed on the study group of 304 people of the Caucasian race, including 155 males and 149 females, who were chosen after sport classification. 

### 2.2. Extraction of Genomic DNA

Saliva was collected with a GeneFiX™ DNA Saliva Collector kit (Isohelix, Maidstone, UK). Using this kit we were able to safely transport biological material without keeping the samples at low temperature. Genomic DNA was extracted with a GeneFiX™ Saliva DNA Isolation Kit (Isohelix, Maidstone, UK) according to the manufacturer’s protocol. The quality and quantification of DNA was assessed using a NanoDrop spectrophotometer (Thermo Scientific, Waltham, MA, USA).

### 2.3. ACTN3 Genotyping

**Figure 1:**
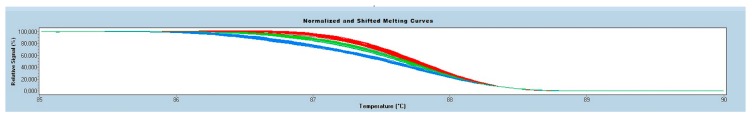
Normalised graph for high-resolution melting (HRM), genotype: RR—red line, RX—blue line and XX—green line.

The genotypes for the *ACTN3* rs1815739 polymorphism were detected with a polymerase chain reaction (PCR) followed by a high-resolution melting (HRM) analysis with a Cobas^®^ 4800 Real-Time PCR System (Roche, Basel, Switzerland). Amplification of the 136-bp fragment of exon 15 was performed with the forward primer 5’-GCACGATCAGTTCAAGGCAAC-3’ and the reverse primer 5’-GAGGGTGATGTAGGGATTGGTG-3’, designed by Grealy et al. with modification [22]. The amplification consisted of 10 min at 95 °C followed by 40 cycles of 95 °C for 10 s, annealing of 60 °C for 10 s, and an extension of 72 °C for 10 s. HRM analysis spanned a temperature range from 70 °C to 95 °C, with a ramp rate of 0.02 °C/s and 25 acquisitions/s. The samples were then cooled at 40 °C for 1 s, and the melting curves were analysed to determine the genotype (Figure 1).

In the polymerase chain reaction-restriction fragment length polymorphism (PCR-RFLP) method, PCR products (136 bp) were digested by Dde1 (New England Biolabs, Ipswich, MA, USA) at 37 °C overnight and then analysed with electrophoresis on a 3% agarose gel. Allele X yielded a fragment of 136 bp, whereas allele R yielded two fragments of 98 and 38 bp.

For each genotype, we established positive controls by using both the original test and the alternative PCR-RFLP determination method. A positive control was then included in all genotyping studies on cohort samples. In addition, in HRM tests, genotyping was performed in duplicate, and more than 15% of the samples were verified with PCR-RFLP. We obtained 100% genotype compliance for all tests.

### 2.4. Statistical Analysis

Categorical variables are presented as numbers followed by the percentages of the respective groups in brackets. The differences between categorical variables were evaluated with Pearson’s chi-square test. Continuous variables are presented as medians followed by interquartile ranges in brackets. The Shapiro-Wilk test was used to assess continuous variable distribution. Due to the non-normal distribution, continuous variables were compared with the Mann-Whitney U-test for two groups or Kruskal-Wallis one-way analysis of variance with additional post-hoc comparisons for three or more groups. A correction for multiple testing was applied (Bonferroni correction). The allele frequencies were tested for Hardy-Weinberg equilibrium with an online calculator at http://www.oege.org/software/hwe-mr-calc.shtml. The Statistica 12.5 PL package (Statsoft, Tulsa, OK, USA) was used for other analyses. *P*-values < 0.05 were considered statistically significant.

## 3. Results

A total of 304 young, healthy adults were genotyped for the distribution of R577X alleles. The overall characteristics of the study group are shown in Table 1.

The percentages for each genotype were as follows: 32.6% RR, 51.3% RX, and 16.1% XX, with an allelic distribution of 58.2% for R and 41.8% for X alleles of *ACTN3*. The genotype distribution for the *ACTN3* gene was in Hardy-Weinberg equilibrium, and the allele frequencies in the sample were p(R) = 0.58 and q(X) = 0.42, *p* > 0.05. The results of the genotype distribution for the *ACTN3* gene and other parameters describing body composition, somatotypes, and factors affecting cardiovascular endurance, are presented in Table 2.

**Figure 2:**
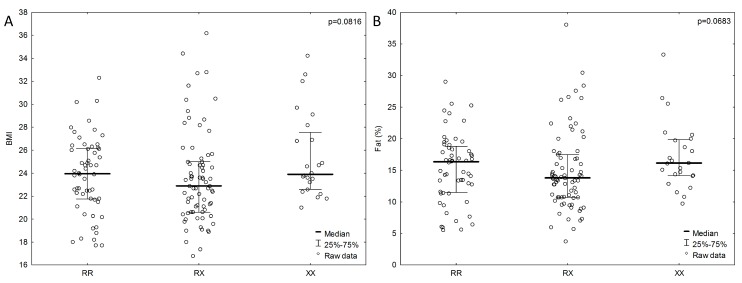
BMI (**A**) and percentage of fat levels (**B**) associated with *ACTN3* gene polymorphism in male group. *P* values from ANOVA (analysis of variance) Kruskal-Wallis test.

We observed no statistically significant relationship between genotypes and body composition in the study group; however, in the male group, a lower percentage of adipose tissue tended to be associated with the *ACTN3* RX genotype than with the XX genotype (RX, *n* = 75; 14.9 ± 6.2 % vs XX, *n* = 24; 17.3 ± 5.3 %; *p* = 0.0683), as also reflected by the BMI for the RX genotype 23.6 ± 4 kg m^−2^ and XX genotype 25.4 ± 3.6 kg m^−2^ (*p* = 0.0816) (Figure 2). In the female group, no such trends were observed. The highest percentage of body fat (RX, *n* = 81; 24.4 ± 6.96 %; *p* = 0.3529) was observed in female subjects with the RX genotype. There were also no differences in the values of indicators used to determine the distribution of body fat (WHR and WHtR) and the *ACTN3* genotypes. 

We also compared the distance run by the participants in the 20 m SRT test. In the male group, individuals with the RR genotype achieved the best results, and those with the XX genotype achieved the worst results (RR, *n* = 56; on average 1590.4 ± 408.2 m vs XX, *n* = 24; 1505.8 ± 478.0 m; *p* = 0.8765). In the female group, the best results were achieved by heterozygotes, and the worst results were achieved by individuals with the XX genotype (RX, *n* = 81; 966 ± 364.31 m vs XX, *n* = 25; 912.8 ± 321.59 m; *p* = 0.8816), similarly to the results for males.

**Figure 3:**
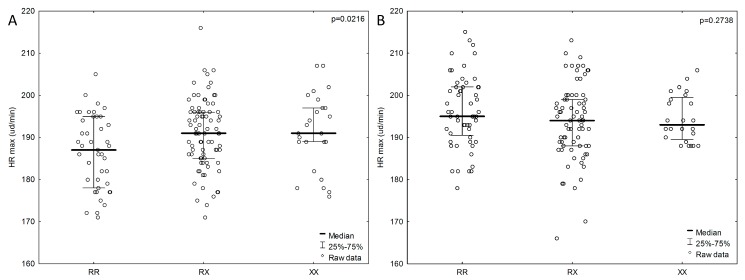
Association of HRmax with *ACTN3* gene polymorphism in the female group (**A**) and male group (**B**). *P* values from ANOVA (analysis of variance) Kruskal-Wallis test.

We also examined the dependence of components affecting cardiopulmonary fitness (VO_2_max and HRmax) on the *ACTN3* genotype. A lower HRmax (RR, *n* = 43; 186.3 ± 8.91 bpm; *p* = 0.0216) was more strongly associated with the RR genotype than with the RX and XX genotypes in the female group, but not in the male group (Figure 3). Male subjects with the RX genotype had lower HRmax values (RX, *n* = 75; 193.7 ± 8.9 bpm; *p* = 0.2738). Males who achieved the best results in the 20 m SRT test (RR, *n* = 56; 1590 ± 408.2 m; *p* = 0.8765) also had higher HRmax values (RR, *n* = 56; 196 ± 8.5 bpm; *p* = 0.2738) and the RR genotype. In the female group, individuals with the RX genotype achieved the best results in the 20 m SRT (RX *n* = 81; 966 ± 364.31 m; *p* = 0.8816), and the HRmax values were average (RX, *n* = 81; 190.9 ± 8.26 bpm; *p* = 0.0216). Male subjects with the XX genotype achieved the poorest results in the physical efficiency index on the basis of VO_2_max (XX, *n* = 24; 51.5 ± 13.4 ml/kg/min; *p* = 0.6908), whereas the female group with the poorest results in the physical efficiency index included individuals with the RX genotype (RX, *n* = 81; 43.9 ± 9.25 ml/kg/min; *p* = 0.5763).

In analysing the relationship between genotypes and somatotypes, we observed differences between males and females. Most of the female subjects with genotypes RR and RX had endomorphic body type, whereas the XX genotype was most frequently represented by females with an ectomorphic body type (Table 3). In contrast, in the male group, all genotypes were most represented in individuals with a mesomorphic body type. A comparison of the male group endomorphs to ectomorphs revealed more individuals with the RR genotype (containing α-actinin-3 in the muscles) in the ectomorph group (Table 3).

## 4. Discussion

The present study investigated the relationship between *ACTN3* gene polymorphism in young, healthy people and the features influencing body structure and cardiorespiratory fitness, and hence performance phenotypes; however, our results did not show the expected significant associations. The *ACTN3* R577X polymorphism has been associated with muscle phenotypes in elite professional and amateur power athletes [4,23]. Some studies report that XX individuals have lower proportions of type II muscle fibres than RR and RX individuals [14,24,25]. In 16–18% of the global human population (XX), there is no α-actinin-3 protein in the skeletal muscle due to homozygosity for a common stop codon polymorphism in the *ACTN3* gene. The α-actinin-3 protein is specifically expressed in the fast-twitch myofibrils responsible for generating force at a high velocity [4,6]. In fact, XX homozygosity in the *ACTN3* “sprinter gene” decreases muscle size, strength, and power but the XX genotype is common in humans [26].

The resulting genotype distribution in our research on the *ACTN3* gene was in Hardy-Weinberg equilibrium. In a study of the Polish population investigating the distribution of *ACTN3* genotypes in professional athletes, the frequencies were RR = 34.5–56.8%; RX = 37.8–49.21%; and XX = 5.4–17.6%, whereas in the control groups, including individuals not practising a professional sport, the frequencies were RR = 35.04–39%; RX = 46.1–50%; and XX = 11–17.6%. Our results are consistent with those of other researchers [6,27,28,29,30,31,32].

We used the 20 m SRT as well as HRmax and VO_2_max measurements to test for cardiorespiratory fitness. The HRmax results in the female group were significant. In our cohort, females with the RR genotype had lower HRmax values (186.3 ± 8.91; *p* = 0.0216), whereas males with the RX genotype had lower HRmax values (193.7 ± 8.9). The opposite observations were reported by Deschamps et al., who found that a significant association of *ACTN3* gene polymorphism (RR vs RX) with peak HR only in males (n = 98), and not in females (*n* = 102) or the combined cohort [26]. In a study of 150 healthy males, Pasqua et al. compared HRmax values and noted that the highest values were in those with the RR genotype [33]. 

Skeletal muscle with a high proportion of fast-twitch fibres consumes more oxygen than the slow-twitch dominant muscle at submaximal stimulation, despite a greater overall oxidative capacity [34]. It is possible that RR genotype-driven differences in fibre type are responsible for differences in submaximal exercise oxygen consumption [35]. In fact, our VO_2_max results were not statistically significant, but Deschamps et al. reported that individuals with the RR vs. XX genotype achieve higher mean VO_2_max peak scores independently of sex (*n* = 200). They reported that *ACTN3* RR homozygotes have up to 15% higher peak oxygen consumption, VO_2_max peak, as compared with XX homozygotes. Higher VO_2_max indicates better functioning of the muscular system in conjunction with the cardiovascular system. Individuals with the XX genotype have a higher adipose tissue content and lower VO_2_max, and thus a greater cardiometabolic risk [26]. In contrast to Deschamps et al., Pimenta et al. suggested that the XX genotype is associated with higher VO_2_max than the RR genotype (male *n* = 200) [36]. Gomez-Gallego et al. observed that RR/RX professional road cyclists exhibit a significantly higher peak power output and ventilatory threshold than their XX counterparts (*n* = 46) [37]. The persistence of the R allele in elite endurance athletes is very important because it reflects the physiological demands of endurance events, in which forceful muscle contractions are essential (e.g., sprint starts and finishes) [38]. There is a close relationship between maximal aerobic capacity and the ability for intense effort [7]. 

We also attempted to correlate body composition parameters, such as BMI, fat content, WHR and WHtR, with *ACTN3* gene polymorphism. A more favourable power-to-weight ratio is observed in individuals with a lower body fat content. Our study showed that males with the XX genotype tended to have higher BMI and body fat content, whereas studies conducted by Moran *et al.* revealed no such tendency (*n* = 525) [15]. The results of Ellis et al. are consistent with our findings, if we consider body fat (%) in the male group (*n* = 211) [39]. Consistent with our results, Moran and Ellis also found no statistically significant differences in any examined group. In the female group observed by Deschamps et al., the highest values for both of these parameters were found in those with genotype XX; however, this result was not confirmed in our findings [26].

For *ACTN3* R577X polymorphism, we found no relationship between the somatotype and genotype; however, Güereca-Arvizuo et al. found that both male and female athletes with the RR genotype have a mesomorphic somatotype, whereas people with the XX genotype are characterized by an ectomorphic somatotype [40].

Most previous studies demonstrated a relationship between the RR genotype and speed. For example, in Moran et al., a speed test was used (40-m dash), and only homozygous RR and RX males covered the distance most quickly [15]. Pimenta et al. reported a similar relationship [36]. Thus, only the R allele and RR genotype are associated with stronger performance in sprinting or power-based events. Yang et al. demonstrated that both male and female elite sprint athletes have significantly higher frequencies of the R allele than individual controls. Thus, the presence of α-actinin-3 has a favourable effect on the functioning of skeletal muscle in generating forceful contractions at high velocity and provides an evolutionary advantage because of increased sprint performance [4]. A similar relationship was presented by Lucia et al. and Niemi et al. [41,42]; however, according to Yang et al., the genotype XX, which leads to α-actinin3 deficiency, is associated with better results in endurance events [4,43]. Furthermore Massidda et al. reported that football players with XX genotypes have a higher risk of muscle injuries than players with RR genotypes [44]. Houweling et al. found that sprint athletes have lower frequencies of genotype XX than control individuals [45]. Although some studies found that endurance athletes have a higher frequency of the XX genotype [46,47,48], other studies found no differences in the genotype frequencies between endurance athletes and controls [38,49,50].

According to Pasqua et al., the *ACTN3* R577X polymorphism may generate different phenotypes, with the R allele supporting muscle force and the X allele being preferred for aerobic metabolism. RX individuals show intermediate muscle endurance, because a lower energy expenditure is required to sustain the same running speed [33]. In contrast, Saunders et al. reported no association of the R577X polymorphism with endurance performance [49]. 

Sports ability is influenced by the sum of factors such as the individual’s genetic profile, gene interactions and related physiology, psychological predisposition, and environmental factors [49]. While a few genes have been repeatedly associated with elite athletic performance, these associations are not predictive, and the use of genetic testing for these variants in talent selection is premature. This type of research further highlights the need to caution against premature commercialization of genetic tests for sports predisposition. 

One limitation of this study is that the group sample size was too low for population studies in genetics research; however, testing a much larger group in the 20 m SRT with direct measurement of VO_2_max would be very difficult to achieve. Another limitation may be that we took into account the polymorphism of only one gene. One gene does not necessarily directly translate into a set of features describing body composition or cardiorespiratory fitness. In contrast, no speed test was planned in our research, and there is a known correlation between *ACTN3* polymorphism and speed. Our future research on the same study group will focus on the analysis of polymorphisms in other genes that may affect the studied traits. 

## 5. Conclusions

On the basis of our research, we observed a possible effect of *ACTN3* gene polymorphism on cardiorespiratory fitness. Females with the RR genotype had a lower HR max, which could indicate better functioning of the circulatory and respiratory system, and better adaptation to physical exercise. Because we observed no such a relationship in the male group, more research should be performed to confirm the effect of *ACTN3* gene polymorphism on cardiorespiratory fitness. In our study, the analysis of *ACTN3* polymorphism did not clearly illustrate the relationships among genotype, body composition, and somatotype in young, healthy adults. Our findings indicate that, on the basis of a single gene, the predisposition to achieve a specific level of sport endurance and sprint power cannot be confirmed. Current evidence suggests that a favourable genetic profile comprising many genes, when combined with appropriate training, may be advantageous for the achievement of elite athletic status.

## Figures and Tables

**Table 1:** Main characteristics of the study group.

	Age (y)	Mass (kg)	Height (cm)	BMI (kg m^−2^)	Distance (m)	HRmax (bpm)	VO_2_max (ml/kg/min)
Female *N* = 149	20.3 ± 1.2	59.3 ± 9.3	165.3 ± 5.7	21.6 ± 3.2	953.6 ± 352.3	189.6 ± 8.8	44.5 ± 8.3
Male *N* = 155	20.7 ± 1.8	75.6 ± 12.1	177.8 ± 6.8	23.9 ± 3.8	1522.6 ± 430.9	194.2 ± 8.4	54.1 ± 8.0

Note: Values are means ± SD. Abbreviations: BMI—body mass index; HRmax—maximal heart rate; VO_2_max—maximum oxygen uptake.

**Table 2:** Genotype distribution and other parameters for the *ACTN3* gene.

Test parameters		Males (*N* = 155RR = 56; RX = 75; XX = 24)	Females (*N* = 149, RR = 43; RX = 81; XX = 25)
Percentage distribution of genotypes (%)	RR	36.13	28.86
	RX	48.39	54.36
	XX	15.48	16.78
BMI (kg m^−2^)	RR	23.7 ± 3.4	21.4 ± 2.59
	RX	23.6 ± 4.0	21.9 ± 3.35
	XX	25.4 ± 3.6	21.1 ± 3.32
*P* value		0.0816	0.5828
Fat (%)	RR	15.3 ± 5.5	22.9 ± 6.44
	RX	14.9 ± 6.2	24.4 ± 6.96
	XX	17.3 ± 5.3	22.3 ± 7.76
*P* value		0.0683	0.3529
Fat (kg)	RR	11.9 ± 5.6	13.7 ± 5.29
	RX	11.9 ± 7.4	15.4 ±7.17
	XX	14.3 ± 6.4	13.5 ± 6.92
*P* value		0.0641	0.3152
WHtR	RR	0.45 ± 0.04	0.42 ± 0.03
	RX	0.45 ± 0.05	0.42 ± 0.04
	XX	0.47 ± 0.05	0.42 ± 0.05
*p* value		0.4247	0.8194
WHR	RR	0.81 ± 0.03	0.72 ± 0.03
	RX	0.83 ± 0.05	0.72 ± 0.04
	XX	0.82 ± 0.04	0.72 ± 0.05
*p* value		0.3160	0.9257
VO_2_max (ml/kg/min)	RR	54.5 ± 8.5	45.6 ± 7.40
	RX	54.8 ± 7.7	43.9 ± 9.25
	XX	51.5 ± 13.4	45.0 ± 6.12
*p* value		0.6908	0.5763
HRmax (bpm)	RR	196.0 ± 8.5	186.3 ± 8.91
	RX	193.7 ± 8.9	190.9 ± 8.26
	XX	194.5 ± 5.5	191.5 ± 8.89
*p* value		0.2738	**0.0218**
Distance (m)	RR	1590.4 ± 408.2	953.0 ± 356.15
	RX	1539.5 ± 429.2	966.0 ± 364.31
	XX	1505.8 ± 478.0	912.8 ± 321.59
*p* value		0.8765	0.8816
Ectomorphy (%)	RR	39.02	29.39
	RX	53.66	46.34
	XX	7.32	24.39
*p* value		0.3752	0.3720
Mesomorphy (%)	RR	35.71	35.29
	RX	44.90	44.12
	XX	19.39	20.59
*p* value		0.3752	0.3720
Endomorphy (%)	RR	35.71	25.81
	RX	57.14	61.29
	XX	7.14	12.90
*p* value		0.3752	0.3720

Note: The specific parameters are characterized by the reported mean ± SD (standard deviation); (N) population size. Abbreviations: BMI—body mass index, WHtR—waist height ratio, WHT—waist hip ratio, VO_2_max—maximum oxygen uptake, HRmax—maximal heart rate. The analysed group was in Hardy-Weinberg equilibrium as the numbers expected (observed) RR, RX and XX cases were 103.06 (99), 147.89 (156), 53.06 (49), respectively This resulted in X allele frequency of 0.58 and q allele frequency of 0.42, respectively *p* > 0.05.

**Table 3:** Distribution of *ACTN3* genotypes according to somatotype (*p* = 0.37).

Sex	Genotypes	Mesomorphy	Endomorphy	Ectomorphy
Males	RR	62.50%	8.93%	28.57%
	RX	59.46%	10.81%	29.73%
	XX	82.61%	4.35%	13.04%
Females	RR	30.00%	40.00%	30.00%
	RX	20.83%	52.78%	26.39%
	XX	28.00%	32.00%	40.00%

## References

[1] Pickering C., Kiely J. (2017). ACTN3: More than Just a Gene for Speed. Front. Physiol..

[2] Fattahi Z., Najmabadi H. (2012). Prevalence of ACTN3 (the athlete gene) R577X polymorphism in Iranian population. Iran. Red Crescent Med. J..

[3] Blanchard A., Ohanian V., Critchley D. (1989). The structure and function of alpha-actinin. J. Muscle Res. Cell Motil..

[4] Yang N., MacArthur D.G., Gulbin J.P., Hahn A.G., Beggs A.H., Easteal S., North K. (2003). ACTN3 genotype is associated with human elite athletic performance. Am. J. Hum. Genet..

[5] Murphy A.C.H., Young P.W. (2015). The actinin family of actin cross-linking proteins—A genetic perspective. Cell Biosci..

[6] North K.N., Yang N., Wattanasirichaigoon D., Mills M., Easteal S., Beggs A.H. (1999). A common nonsense mutation results in alpha-actinin-3 deficiency in the general population. Nat. Genet..

[7] Broos S., Malisoux L., Theisen D., van Thienen R., Ramaekers M., Jamart C., Deldicque L., Thomis M.A., Francaux M. (2016). Evidence for ACTN3 as a Speed Gene in Isolated Human Muscle Fibers. PLoS ONE.

[8] Clarkson P.M., Devaney J.M., Gordish-Dressman H., Thompson P.D., Hubal M.J., Urso M., Price T.B., Angelopoulos T.J., Gordon P.M., Moyna N.M. (2005). ACTN3 genotype is associated with increases in muscle strength in response to resistance training in women. J. Appl. Physiol..

[9] Guth L.M., Roth S.M. (2013). Genetic influence on athletic performance. Curr. Opin. Pediatr..

[10] Surninaga R., Matsuo M., Takeshima Y., Nakamura H., Wada H. (2000). Nonsense mutation of the alpha-actinin-3 gene is not associated with dystrophinopathy. Am. J. Med. Genet..

[11] Druzhevskaya A.M., Ahmetov I.I., Astratenkova I.V., Rogozkin V.A. (2008). Association of the ACTN3 R577X polymorphism with power athlete status in Russians. Eur. J. Appl. Physiol..

[12] Papadimitriou I.D., Papadopoulos C., Kouvatsi A., Triantaphyllidis C. (2008). The ACTN3 gene in elite Greek track and field athletes. Int. J. Sports Med..

[13] Santiago C., Gonzalez-Freire M., Serratosa L., Morate F.J., Meyer T., Gomez-Gallego F., Lucia A. (2008). ACTN3 genotype in professional soccer players. Br. J. Sports Med..

[14] Vincent B., De Bock K., Ramaekers M., Van den Eede E., Van Leemputte M., Hespel P., Thomis M.A. (2007). ACTN3 (R577X) genotype is associated with fiber type distribution. Physiol. Genomics.

[15] Moran C.N., Yang N., Bailey M.E.S., Tsiokanos A., Jamurtas A., MacArthur D.G., North K., Pitsiladis Y.P., Wilson R.H. (2007). Association analysis of the ACTN3 R577X polymorphism and complex quantitative body composition and performance phenotypes in adolescent Greeks. Eur. J. Hum. Genet..

[16] Leger L.A., Mercier D., Gadoury C., Lambert J. (1988). The multistage 20 metre shuttle run test for aerobic fitness. J. Sports Sci..

[17] World Health Organization (WHO) (2011). Waist Circumference and Waist-hip Ratio: Report of a WHO Expert Consultation, Geneva, 8–11 December 2008.

[18] Carter J.E.L. The Heath-Carter Anthropometric Somatotype-Instruction Manual. htth/cmvwsomatotypeorg/Heath—CarterManual.pdf.

[19] Rahmawati N.T., Hastuti J., Ashizawa K. (2004). Growth and somatotype of urban and rural Javanese children in Yogyakarta and Bantul, Indonesia. Anthropol. Sci..

[20] Carter J.E.L., Heath B.H. (1990). Somatotyping Development Applications.

[21] Tóth T., Michalíková M., Bednarčíková L., Živčák J., Kneppo P. (2014). Somatotypes in sport. Acta Mech. Autom..

[22] Grealy R., Herruer J., Smith C.L.E., Hiller D., Haseler L.J., Griffiths L.R. (2015). Evaluation of a 7-Gene Genetic Profile for Athletic Endurance Phenotype in Ironman Championship Triathletes. PLoS ONE.

[23] Weyerstrass J., Stewart K., Wesselius A., Zeegers M. (2018). Nine genetic polymorphisms associated with power athlete status - A Meta-Analysis. J. Sci. Med. Sport.

[24] Pereira A., Costa A.M., Leitao J.C., Monteiro A.M., Izquierdo M., Silva A.J., Bastos E., Marques M.C. (2013). The influence of ACE ID and ACTN3 R577X polymorphisms on lower-extremity function in older women in response to high-speed power training. BMC Geriatr..

[25] Broos S., Malisoux L., Theisen D., Francaux M., Deldicque L., Thomis M.A. (2012). Role of alpha-actinin-3 in contractile properties of human single muscle fibers: A case series study in paraplegics. PLoS ONE.

[26] Deschamps C.L., Connors K.E., Klein M.S., Johnsen V.L., Shearer J., Vogel H.J., Devaney J.M., Gordish-Dressman H., Many G.M., Barfield W. (2015). The ACTN3 R577X Polymorphism Is Associated with Cardiometabolic Fitness in Healthy Young Adults. PLoS ONE.

[27] Papadimitriou I.D., Lockey S.J., Voisin S., Herbert A.J., Garton F., Houweling P.J., Cieszczyk P., Maciejewska-Skrendo A., Sawczuk M., Massidda M. (2018). No association between ACTN3 R577X and ACE I/D polymorphisms and endurance running times in 698 Caucasian athletes. BMC Genom..

[28] Fedovskaya O., Cieszczyk P., Leońska-Duniec A., Buryta M., Grenda A., Wiażewicz A. (2012). Role and Importance of the R577X Polymorphism in the ACTN3 Gene in High Elite Polish Rowers.

[29] Fedotovskaya O., Eider J., Cieszczyk P., Ahmetov I., Moska W., Sawczyn S., Ficek K., Leonska-Duniec A., Maciejewska-Karlowska A., Sawczuk M. (2013). Association of muscle-specific creatine kinase (CKM) gene polymorphism with combat athlete status in Polish and Russian cohorts. Arch. Budo.

[30] Grenda A., Leonska-Duniec A., Kaczmarczyk M., Ficek K., Krol P., Cieszczyk P., Zmijewski P. (2014). Interaction Between ACE I/D and ACTN3 R557X Polymorphisms in Polish Competitive Swimmers. J. Hum. Kinet..

[31] Orysiak J., Busko K., Michalski R., Mazur-Rozycka J., Gajewski J., Malczewska-Lenczowska J., Sitkowski D., Pokrywka A. (2014). Relationship between ACTN3 R577X polymorphism and maximal power output in elite Polish athletes. Med. Lith..

[32] Cieszczyk P., Eider J., Ostanek M., Arczewska A., Leonska-Duniec A., Sawczyn S., Ficek K., Krupecki K. (2011). Association of the ACTN3 R577X Polymorphism in Polish Power-Orientated Athletes. J. Hum. Kinet..

[33] Pasqua L.A., Bueno S., Matsuda M., Marquezini M.V., Lima-Silva A.E., Saldiva P.H.N., Bertuzzi R. (2016). The genetics of human running: ACTN3 polymorphism as an evolutionary tool improving the energy economy during locomotion. Ann. Hum. Biol..

[34] Kushmerick M.J., Meyer R.A., Brown T.R. (1992). Regulation of oxygen-consumption in fast-twitch and slow-twitch muscle. Am. J. Physiol..

[35] Shah B.N. (2013). On the 50th anniversary of the first description of a multistage exercise treadmill test: Re-visiting the birth of the ‘Bruce protocol’. Heart.

[36] Pimenta E.M., Coelho D.B., Veneroso C.E., Barros Coelho E.J., Cruz I.R., Morandi R.F., Pussieldi G.D., Carvalho M.R., Garcia E.S., De Paz Fernandez J.A. (2013). Effect of ACTN3 gene on strength and endurance in soccer players. J. Strength Cond. Res..

[37] Gomez-Gallego F., Santiago C., Gonzalez-Freire M., Muniesa C.A., del Valle M.F., Perez M., Foster C., Lucia A. (2009). Endurance Performance: Genes or Gene Combinations?. Int. J. Sports Med..

[38] Ahmetov I.I., Druzhevskaya A.M., Astratenkova I.V., Popov D.V., Vinogradova O.L., Rogozkin V.A. (2010). The ACTN3 R577X polymorphism in Russian endurance athletes. Br. J. Sports Med..

[39] Ellis L., Collins C., Brown J., Pooley W. (2017). Is AGT The New Gene For Muscle Performance? An Analysis of AGT, ACTN3, PPARA and IGF2 on Athletic Performance, Muscle Size and Body Fat Percentage in Caucasian Resistance Training Males. J. Athl. Enhanc..

[40] Güereca-Arvizuo J., Ramos-Jiménez A., Flores-Martínez N., Reyes-Leal G., Hérnandez-Torres R.P. (2017). ACTN3 genotypes and their association with athletes somatotype: Results of a pilot study. ECORFAN Ecuad. J..

[41] Lucia A., Gomez-Gallego F., Santiago C., Bandres F., Earnest C., Rabadan M., Alonso J.M., Hoyos J., Cordova A., Villa G. (2006). ACTN3 genotype in professional endurance cyclists. Int. J. Sports Med..

[42] Niemi A.K., Majamaa K. (2005). Mitochondrial DNA and ACTN3 genotypes in Finnish elite endurance and sprint athletes. Eur. J. Hum. Genet..

[43] Pasqua L.A., Bueno S., Artioli G.G., Lancha A.H., Matsuda M., Marquezini M.V., Lima-Silva A.E., Saldiva P.H.N., Bertuzzi R. (2016). Influence of ACTN3 R577X polymorphism on ventilatory thresholds related to endurance performance. J. Sports Sci..

[44] Massidda M., Voisin S., Culigioni C., Piras F., Cugia P., Yan X., Eynon N., Calo C.M. (2019). ACTN3 R577X Polymorphism Is Associated with the Incidence and Severity of Injuries in Professional Football Players. Clin. J. Sport Med. Off. J. Can. Acad. Sport Med..

[45] Houweling P.J., Papadimitriou I.D., Seto J.T., Perez L.M., Del Coso J., North K.N., Lucia A., Eynon N. (2018). Is evolutionary loss our gain? The role of ACTN3 p.Arg577Ter (R577X) genotype in athletic performance, ageing, and disease. Hum. Mutat..

[46] Ruiz J.R., Santiago C., Yvert T., Muniesa C., Diaz-Urena G., Bekendam N., Fiuza-Luces C., Gomez-Gallego F., Femia P., Lucia A. (2013). ACTN3 genotype in Spanish elite swimmers: No “heterozygous advantage”. Scand. J. Med. Sci. Sports.

[47] Eynon N., Duarte J.A., Oliveira J., Sagiv M., Yamin C., Meckel Y., Goldhammer E. (2009). ACTN3 R577X Polymorphism and Israeli Top-level Athletes. Int. J. Sports Med..

[48] Ruiz J.R., Gomez-Gallego F., Santiago C., Gonzalez-Freire M., Verde Z., Foster C., Lucia A. (2009). Is there an optimum endurance polygenic profile?. J. Physiol. Lond..

[49] Saunders C.J., September A.V., Xenophontos S.L., Cariolou M.A., Anastassiades L.C., Noakes T.D., Collins M. (2007). No association of the ACTN3 gene R577X polymorphism with endurance performance in ironman triathlons. Ann. Hum. Genet..

[50] Doring F.E., Onur S., Geisen U., Boulay M.R., Perusse L., Rankinen T., Rauramaa R., Wolfahrt B., Bouchard C. (2010). ACTN3 R577X and other polymorphisms are not associated with elite endurance athlete status in the Genathlete study. J. Sports Sci..

